# Serious Neurological Adverse Events after Ivermectin—Do They Occur beyond the Indication of Onchocerciasis?

**DOI:** 10.4269/ajtmh.17-0042

**Published:** 2017-12-04

**Authors:** Rebecca E. Chandler

**Affiliations:** Uppsala Monitoring Centre, Uppsala, Sweden

## Abstract

Serious neurological adverse events have been reported from large scale community-based ivermectin treatment campaigns against *Onchocerciasis volvulus* in Africa. The mechanism of these events has been debated in the literature, largely focusing on the role of concomitant infection with *Loa loa* versus the presence of mdr-1 gene variants in humans allowing ivermectin penetration into the central nervous system. A case series of serious neurological adverse events occurring with the use of ivermectin outside of the onchocerciasis indication has been identified in VigiBase, an international database of suspected adverse drug reactions. Forty-eight cases have been reported from multiple countries in which ivermectin has been prescribed for multiple indications; clinical review excluded 20 cases with more probable explanations or other exclusion criteria. Within the remaining 28 cases, there is supportive evidence for a causative role of ivermectin including presence of the drug in brain tissue in one case and recurrence of symptoms on repeated exposure in three cases. This series suggests that serious neurological adverse events observed with the use of ivermectin in the treatment of onchocerciasis may not be entirely explained by concomitant high burden loiasis infections. By comparison with the extensive post marketing experience with ivermectin in the successful treatment of parasitic infections, the number of reported cases suggests that such events are likely rare. However, elucidation of individual-level risk factors could contribute to therapeutic decisions that can minimize harms. Further investigation into the potential for drug–drug interactions and explorations of polymorphisms in the mdr-1 gene are recommended.

## INTRODUCTION

Ivermectin is a member of the class of avermectins, which are highly active broad-spectrum, anti-parasitic agents. It is indicated for use in the treatment of strongyloidiasis (*Strongyloides stercoralis*) and onchocerciasis (*Onchocerca volvulus*).^1^ It is also commonly used to treat scabies, although usage patterns for this indication vary between countries. In the scabies indiciation, it is approved for first-line therapy in some countries (within Europe), second-line therapy in others (such as Australia), or only recommended for special circumstances, such as immunocompromised or institutionalized patients or when topical therapy has failed (the United States).^2–4^ Furthermore, it has been suggested for use in the treatment of head lice given the recent increase in the burden of this condition in the developed world.^5^ Ivermectin is not thought to readily cross the blood-brain barrier in humans as it is excluded by a P-glycoprotein drug pump (mdr-1).^6^ Therefore, it has been considered to be free of the potential to cause neurological adverse drug reactions, except in situations of overdose.^7,8^

Serious neurological adverse events were initially reported in public health programs in Africa to eliminate onchocerciasis through community-based ivermectin treatment; cases of encephalopathy and coma were reported in Cameroon and the Democratic Republic of Congo in persons who concomitantly harbored high densities of another filarial species, *Loa loa*. Subsequent analyses revealed a correlation between pre-ivermectin treatment *L. loa* microfilarial density and the risk of developing a serious neurological adverse event.^9,10^ The mechanism of these events has been debated in the literature, largely focusing on the role of concomitant high burden infection with *L. loa* versus the presence of mdr-1 gene variants in humans allowing ivermectin penetration into the central nervous system (CNS).^11,12^

Statistical signal detection screening of the VigiBase, a global database of individual case safety reports (reports) of adverse events for the WHO program for International Drug Monitoring, is performed on a periodic basis. During a screening designed to be sensitive to reports from Africa, Asia, Latin America, and the Caribbean, a serious report of ataxia from the Democratic Republic of Congo was identified which stated: “This patient …realises these conditions [encephalopathy]. It is very surprising to notice that there were no microfilariae in the calibrated thick blood smear.” The lack of evidence of a high density of *L. loa* in the blood smear appeared to challenge the literature on serious neurological adverse events after ivermectin, and it therefore triggered a review of all reports of such events for ivermectin beyond the indication of use for onchocerciasis.

## METHODS

The data source was VigiBase, a computerized pharmacovigilance system in which reports describing adverse events are recorded in a structured, hierarchical form. Reports are received from national pharmacovigilance centers in the 125 countries participating in the WHO Program for International Drug Monitoring.^13^ All reports for ivermectin received into VigiBase up to November 27, 2016, were identified for use in this investigation. All analyses were performed on adverse event data using the Medical Dictionary for Regulatory Activities (MedDRA^®^) System Organ Class (SOC) and Preferred Term-level terminology.

## RESULTS

A total of 1,668 reports for ivermectin were identified. The most commonly reported adverse events for ivermectin were pruritus (25.3%), headache (13.9%), and dizziness (7.5%). Under the MedDRA SOC “Neurological disorders,” there were a total of 426 reports; 156 of these were classified as “serious” according to ICH Guidance.^14^ Of the serious reports, 60.9% (95) originated from Africa, 20.5% (32) from the Americas, 12.2% (19) from Europe, and 6.4% (10) from Asia. One duplicate report was identified and excluded from the analysis.

Sixty-four of the 155 serious reports described the use of ivermectin for *O. volvulus*. Forty-two reports did not include an indication; one reported only “infection parasitic.” The remaining 48 reports underwent clinical review, and twenty reports were further excluded from this analysis. Reasons for exclusion were neurological adverse events reported in the context of other clinical syndromes (lactic acidosis/circulatory collapse, cerebral infarction/cerebral artery embolism, neuroleptic malignant syndrome, hepatitis/hepatic failure, brain cancer, pneumonia with hypotension, accidental exposure to product, sepsis complicating chemotherapy, multi-organ failure, history of epilepsy, and Alzheimer’s disease), topical ivermectin for rosacea, prolonged time to onset of events in comparison with the known half-life of ivermectin (14 days and 8 years), and unclear onset of symptoms in relation to ivermectin.

The remaining 28 reports are included in this case series (Table 1). The cases were received from the United States, France, Japan, the Netherlands, Germany, Canada, and Sierra Leone. The patient ages were included in 25 reports and ranged from 11 to 97 years. Fourteen reports described adverse events in males, 13 in females, and the gender was not provided in one report. Scabies was included as an indication in 10 reports, acarodermatitis (within the MedDRA terminology, acarodermatitis may be used to indicate any of the following terms: acarodermatitis, Norwegian scabies, *Sarcoptes scabeii* infestation, scabies, and scabies infestation) in eight, filariasis due to *Wucheria bancrofti* in five, strongyloidiasis in three, teniasis in one, and myiasis in one. The time to onset of the serious neurological events ranged from hours to 7 days, with 14 cases noting a time to onset of 1 day or less. Examples of serious neurological adverse events reported included such terms as unable to walk, consciousness disturbed or depressed level of consciousness or loss of consciousness, seizure or convulsion, encephalopathy or coma, and tremor. The reported dosages of ivermectin ranged between 3 and 24 mg. Most of the cases reported a one-time dose or two doses separated by 1 week. Weight information was provided for most of the cases, and there was no suggestion of overdose based on the data provided. Nine reports documented a positive dechallenge, with resolution of symptoms after discontinuing ivermectin without further intervention. Three reports documented a positive rechallenge, with recurrence of symptoms with re-exposure to ivermectin, including one case with repeated symptoms on three separate treatment courses with ivermectin. Concomitant medications were reported in 20 cases. In nine of the cases, the drugs co-administered with ivermectin were also reported to be “suspected” for the described adverse drug reactions, including oxatomide, piperonyl butoxide/esdepallethrime (topical), darunavir and ritonavir, terbinafine, and albendazole. Eight cases reported concomitant drugs with known CNS effects, including antidepressants, antipsychotics, benzodiazepines, and anticonvulsants; however, in none of these cases were the concomitant CNS-acting agents considered to be “suspected.”

**Table 1:** Case series describing serious neurological adverse events after treatment with ivermectin beyond the onchocerciasis indication

Case	Age/sex	Indication	Dose	Weight (kg)	Other suspect (S) or concomitant (C) medications	Reported adverse event terms	Time to onset	Additional info
1	11/F	Scabies	9 mg	40	–	Encephalopathy, coma, emesis	1 day	Recovered. Positive dechallenge. Presented with depressed level of consciousness, without evidence of fever or hypoglycaemia. Glasgow coma scale 9. Naloxone given without effect. LP, EEG, and MRI all performed and negative. Multiple cultures and serologies negative.
2	28/M	Scabies	18 mg	–	–	Confusional state, amnesia, malaise, emesis	1 day	Recovered. Similar symptoms reported twice in the past after ivermectin. Presented with confusional state, Glasgow coma scale 13. Neurological exam negative.
3†	24/M	Scabies	3 mg	–	Oxatomide (S)	Confusion, convulsive disorder cephalgia, fatigue, fall	0 day	Recovered. Patient presented with headache, fatigue and fall. Sent to hospital. Sleepy but awake, opened eyes to pain. No evidence of tongue biting, urinary incontinence. Normal neurological exam, normal CT. No previous history of neurological disease.
4	18/M	Scabies infestation	15 mg	79	–	Lightheadedness, headache, unable to walk	1 day	“Patient developed headache and dizziness to the point of being unable to walk.” Positive dechallenge Recovered in 24 hours.
5	32/F	Scabies	24 mg	109	–	Tremor, dizzy spells, mucosal dryness, abdominal pain lower	8 hours	–
6	14/M	Scabies	1 DF*, 1 per 1 day	–	Piperonyl butoxide/esdepallethrime (topical) (S)	Dizziness, crying abnormal, monoparesis, tremor, rigors, chills	10 hours	Recovered. Positive dechallenge
7	48/F	Scabies	9 mg day 0, 9 mg day 7	68	Piperonyl butoxide/esdepallethrime (topical) (S)	Muscle weakness, hypoaesthesia, paraesthesia	–	Recovered. Symptoms occurred after each dose of ivermectin and topical piperonyl butoxide. Lasted 20–40 minutes. Was unable to speak.
8	33/M	Scabies	12 mg	65	Darunavir, ritonavir (both S)	Convulsions generalized	1 day	Recovered. Positive dechallenge with all 3 drugs. Patient had started darunavir 12 months prior and ritonavir 8 days prior. Head scan normal.
9†	–/M	Scabies infestation	12 mg	70	Ranitidine, amantidine, trazadone, lorazepam, haloperidol, toprimate, hydroxyzine, risperidone (all C)	Confusional state, unconsciousness	–	–
10	81/M	Scabies	3 mg	–	–	Cerebellar syndrome, mental confusion, MRI abnormal	1–2 days	Drug withdrawn, no effect observed. Patient with altered state of consciousness, aggression, confusion. Exam revealed thrombocytopenia, eosinophilia, dehydration and renal failure. MRI abnormal 2 weeks after dosing.
11†	58/F	Acarodermatitis	12 mg	60	Alprazolam, Etizolam (both C)	Consciousness disturbed	0 day	Positive dechallenge. Recovered within 8 days. No dates of use for alprazolam or etizolam provided.
12†	51/M	Acarodermatitis	18 mg day 0, 18 mg day 7	79	Pregabalin, lamotrigine, aripiprazole, meloxicam, simvastatin, docusate (all C)	Abasia, aphasia, blindness, disease recurrence	–	Positive rechallenge
13	54/F	Acarodermatitis	Two “pills” day 0, two “pills” day 7	68	–	Convulsion, local swelling, nausea, headache, heart rate increased, confusional state	–	–
14	81/M	Acarodermatitis	9 mg	50	Rivastigmine, memantine, lornoxicam, troxipide (all C)	Tremor, pyrexia	0 day	Positive dechallenge
15†	–/–	Acarodermatitis	–	–	Valproic acid, levetiracetam (both C)	Seizure, off label use	–	No dates of use provided for any of the reported medicines. Only ivermectin reported as “suspected.”
16	81/F	Acarodermatitis	12 mg day 0, 12 mg day 7	–	Digoxin, rebamipide, crotamiton, magnesium oxide, senna (all C)	Depressed level of consciousness, vomiting, asphyxia, pruritus aggravated, skin eruption	5 days after last dose	Died, 5 days after last dose from the events of depressed level of consciousness and asphyxia. Digoxin initiated 1 day prior to death.
17†	56/F	Acarodermatitis	12 mg	55	Terbinafine (S), dexlansoprazole, milnacipran, gabapentin, promethazine, meloxicam, trazadone, levothyroxine, propranolol, Lisinopril, predinose, azathioprine, diazepam, nortriptyline, lansoprazole, amoxicillin, furosemide, hydrochloroquine, vitamin D, vitamins (all C)	Aphasia, somatic delusion abnormal faeces, alopecia, dry mouth, dyspnoea, ear infection, flushing, gastrointestinal motility disorder, headache, heart rate increased, lip swelling, musculoskeletal discomfort, oral discomfort, red blood cell count decreased, swollen tongue, urine color abnormal, urine odor abnormal, weight decreased white blood cell count decreased	2–5 days for aphasia and somatic delusion	Drug withdrawn, no effect observed
18	97/F	Acarodermatitis	9 mg day 0, 9 mg day 7	47	Febuxostat, furosemide, lansoprazole, sennoside a + b, magnesium oxide, carbocisteine, etizolam (all C)	Depressed level of consciousness, loss of consciousness, vomiting	6 days after 1st dose and 5 days after 2nd dose	Recovered. Positive dechallenge
19	64/M	Strongyloidiasis	12 mg oral then subcutaneous	57	–	Coma, neurotoxicity	–	Drug withdrawn, fatal outcome. Tx initiated for *Strongyloides* stercoralis infection in patient on prednisone for giant cell arteritis. Patient was s/p aortic valve replacement. Ivermectin levels measured in brain tissue at autopsy (30 ng/g). None of the most common polymorphisms in mdr-1 present.
20†	59/F	Strongyloidiasis Nematodiasis	21 mg day 0, 21 mg day 1	100	Levothyroxine, olopatadine, vitamines, omega-3, melatonin, ascorbic acid, formoterol/budesonide doxycycline, potassium citrate, pioglitazone, probiotics, vitamin D, prasterone, progesterone, colesevelam, montelukast. desvenlafaxine (all C)	Pain in jaw, tremor chest pain, chills back pain, tachycardia, dyspnoea, loss of consciousness, pain in extremity, thinking abnormal, peripheral coldness, hypersomnia, dizziness, asthenia, feeling abnormal, palpitations, paraesthesia, fatigue, blood potassium decreased, dysgeusia, constipation, muscle twitching, sedation, vertigo, sensation of heaviness, feeling cold, mood altered, feeling drunk, oropharyngeal pain, coxsackie virus test positive, inappropriate schedule of drug administration, orthostatic hypotension, neuralgia,affect lability, hypertension, asthma, confusional state, cough, nystagmus, headache, pyrexia, somnolence	1–2 days	Drug withdrawn, no effect observed.
21	–/M	Strongyloidiasis	18 mg day 0, 18 mg day 1	86	–	Quality of life decreased, sleep disorder	–	–
22	28/M	Filariasis due to Wuchereria bancrofti	12 mg	–	Albendazole (S), diclofenac, amoxicillin (both C)	Unconsciousness	0 day	Recovered. Gastric lavage. Patient concomitantly treated for Ascariasis
23	36/M	Filariasis due to Wuchereria bancrofti	12 mg	–	Albendazole (S)	Headache, vomiting, diarrhea, abdominal discomfort	1 day	Recovered Patient concomitantly treated for Ascariasis
24	43/F	Filariasis due to Wuchereria bancrofti	9 mg	–	Albendazole (S)	Headache, dizziness, vomiting	2 days	Recovered Patient concomitantly treated for Ascariasis
25	11/F	Filariasis due to Wuchereria bancrofti	9 mg	–	Albendazole (S)	Headache, Dizziness, Vomiting	0 day	Recovered Patient concomitantly treated for Ascariasis
26	72/M	Filariasis due to Wuchereria bancrofti	12mg	–	Albendazole (S)	Headache, abdominal discomfort, itching vomiting, oedema	0 day	Recovered Patient concomitantly treated for Ascariasis
27	–/F	Myiasis	12 mg	–	–	Seizure, off label use	–	Not recovered
28	75/F	Taeniasis	6 mg	59	Lisinopril, amlodipine, metoprolol, clopidogrel (all C)	Asthenia, dizziness, dyspnoea, paraesthesia, vision decreased	0 day	Recovered with sequelae

EEG = electroencephalogram; LP = lumbar puncture; MRI = magnetic resonance imaging.

* DF = dosage form; for ivermectin, 1 dosage form = 3 g.

† Cases with concomitant medications with central nervous system effects.

Two patients had a fatal outcome reported. One died of asphyxia 5 days after a dose of ivermectin (case 16). The other fatal case has been previously published and documented the presence of ivermectin in brain tissue: “A 64-year-old male with a past medical history of giant cell arteritis, treated with prednisone developed sepsis, complicated by multisystem organ failure, after an aortic valve replacement. Sputum culture revealed *S. stercoralis*. A diagnosis of *S. stercoralis* hyperinfection syndrome was made; he was initiated on ivermectin 12 mg every 48 hours. He received three oral doses followed by two subcutaneous doses. In spite of clinical and microbiological improvement, the patient remained in a vegetative state and died on day 25. Autopsy revealed an elevated level of ivermectin in the brain tissue, 14 days after the last dose”^15^ (case 19).

## DISCUSSION

From a global database of suspected adverse drug reactions we have identified a case series describing serious neurological adverse events with the use of ivermectin beyond its indication for *O. volvulus*. Supportive evidence for a causative role of ivermectin was found with the identification of ivermectin in brain tissue in one case and recurrence of symptoms on repeated exposure to ivermectin in three cases. This case series suggests that the serious neurological adverse events observed with the use of ivermectin in the treatment of onchocerciasis cannot be entirely explained by concomitant high burden loiasis infections.

Ivermectin exhibits poor penetration of the blood-brain barrier of vertebrate animals due to the presence of a drug-transporting p-glycoprotein.^6^ However, studies in knockout mice for the p-glycoprotein encoding gene, mdr-1, displayed levels of ivermectin in the brain which were 90-fold greater than normal mice.^16^ Furthermore, it is well established in the veterinary world that certain breeds of dogs, such as collies, are sensitive to the neurotoxic effects of ivermectin as a loss of function in the mdr-1 gene in these breeds allows for an accumulation of ivermectin within the brain.^17^ Symptoms of neurotoxicity include lethargy, drooling, tremors/seizures, inability to stand, disorientation, and coma.

Serious neurological events in humans, such as encephalopathy, confusion, stupor, or coma, after ivermectin were initially observed in campaigns to treat *O. volvulus* in African countries. Co-infection with *L. loa* was found to be a risk factor for the development of these reactions, and the product label recommends posttreatment follow up for patients who have been in *L. loa* endemic areas of West and Central Africa.^9,10^ Furthermore, there have also been published concerns raised regarding the safety of use of ivermectin in the treatment of elderly patients with scabies.^18,19^ A study investigating escalating high doses of ivermectin in healthy adults was performed to explore the safety of its use in the treatment of head lice. The authors documented no evidence of CNS toxicity in doses up to 10 times the highest FDA-approved dose of 200 µg/kg. However, the study population was limited to a total of 68 subjects, almost 90% of which were of Hispanic origin.^20^ Drug safety surveillance for idiosyncratic reactions has been recommended.^19^

The product label for ivermectin notes that the neurological events of dizziness (2.8%), somnolence (0.9%), vertigo (0.9%), and tremor (0.9%) were observed in human clinical trials for the treatment of strongyloidiasis and assessed as at least possibly related to ivermectin, whereas drug-related headache (0.2%) was observed in trials for onchocerciasis. The label further includes warnings for the occurrence of serious neurological adverse events in the contexts of concomitant infection of onchocerciasis and loiasis and accidental intoxication with veterinary formulations of ivermectin.^1^ Although some of the adverse events experienced by subjects in this case series were observed in clinical trials (dizziness, headache), there were other events of a more serious nature which are suggestive of ivermectin penetration into the brain: loss of consciousness/depressed level of consciousness, abasia, tremor, vomiting, and coma.

Clinical review of the cases within this case series has focused largely on three important confounders: co-administered drugs with known CNS effects, overdosing, and evidence of secondary impairment of the blood-brain barrier. A number of cases report co-administered drugs with known CNS-effects, such as antihistamines (case 3), antidepressants/antipsychotics (cases 9 and 20), anxiolytics, and/or antiepileptics (cases 9, 11, 12, 15, and 17). In only case 3 was the concomitant drug reported as “suspected” as a potential cause of the reported adverse events. Further assessment of the other cases is complicated by lack of information on the treatment courses of drugs which were not “suspected.” However, in one case (case 11) the symptoms apparently resolved when ivermectin was discontinued (“positive dechallenge”), and in another (case 12) the events recurred with repeated dosing of ivermectin (“positive rechallenge”). Five cases noted concomitant administration of albendazole whose package insert describes both headache and dizziness as possible adverse drug reactions (cases 22–26); in all cases, albendazole was considered also to be “suspected.” In case 22, however, the reported adverse event of “unconsciousness” would not be an expected event from this additional anti-parasitic agent. There was no evidence of overdosing in any of the cases upon review of dosage recommendations and weight data provided in the case reports. Obvious evidence of blood-brain barrier insufficiency, such as sepsis or malignancy, resulted in exclusion from the final case list; however, it is possible that there remain cases in the case series in which the blood-brain barrier has been weakened by the indication for ivermectin, such as strongyloidiasis (case 19).

A number of cases included in the final case series may be related to drug–drug interactions. Drugs that are substrates of CYP3A4 enzymes are often also substrates for P-glycoprotein transport, and thus there may be a risk of increased absorption past the blood-brain barrier with concomitant ivermectin administration.^21^ Several cases presented here reported concomitant use of such drugs, such as statins (case 12), HIV protease inhibitors (case 8), calcium channel blockers (case 28), and benzodiazepines (cases 9, 11, 17, 18). A recent publication documents evidence of an in vitro interaction of ivermectin and a number of antiretroviral agents.^22^ Current labeling for ivermectin contains no warning for co-administration with CYP3A4 substrates.

Another possible explanation is that some humans experiencing serious neurological adverse events after ivermectin therapy may have mutations in the mdr-1 gene, allowing for penetration of ivermectin into the CNS. More than 50 naturally occurring single nucleotide polymorphisms (SNPs) have been identified in the mdr-1 gene; the majority of these SNP are silent, and there is no current evidence of a mutation that results in loss of function. However, various combinations of these SNPs, composing different P-glycoprotein haplotypes, have been found to exhibit reduced mdr-1 expression.^23^ Bourguinat et al.^24^ analyzed mdr-1 genotypes in 13 subjects from Cameroon: four who experienced a serious adverse event and nine who did not. Haplotypes associated with altered drug disposition were present as homozygotes in two of the patients experiencing serious adverse events and in none of the control patients. One of the cases in our series was investigated for the most common polymorphisms associated with decreased mdr-1 expression and found that none were present; however, further details were not provided.^15^

Limitations of spontaneous adverse event reports are that they are come from both voluntary and regulated sources, a suspicion that the reported drug caused the event may or may not be present, and the amount of information included in the report is variable. Detection of “signals” in these types of databases is intended to be hypothesis-generating rather than evidence of causality.

In conclusion, there is evidence that serious neurological adverse events can occur with ivermectin beyond the treatment of *O. volvulus* complicated by concomitant high burden *L. loa* infection. Potential explanations include concomitantly administered drugs which inhibit CYP3A4 and polymorphisms in the mdr-1 gene. By comparison with the extensive post marketing experience with ivermectin in the successful treatment of parasitic infections, the total number of reported cases suggests that such events are likely rare. However, elucidation of individual-level risk factors could contribute to therapeutic decisions that can minimize harms. Further investigation into the potential for drug interactions and explorations of polymorphisms in the mdr-1 gene are recommended.
